# Effects of Donepezil and Medroxyprogesterone Versus Placebo on Weaning in Adult Patients With Non-Pulmonary Etiologies Receiving Invasive Mechanical Ventilation: A triple‐blind Randomized Clinical Trial

**DOI:** 10.3389/fphar.2021.735594

**Published:** 2021-12-06

**Authors:** Zahra Alizadeh, Adeleh Sahebnasagh, Navid Hadadzadegan, Farhad Mohammadi, Fatemeh Saghafi

**Affiliations:** ^1^ Pharmaceutical Sciences Research Center, School of Pharmacy, Shahid Sadoughi University of Medical Sciences and Health Services, Yazd, Iran; ^2^ Clinical Research Center, Department of Internal Medicine, School of Medicine, North Khorasan University of Medical Science, Bojnurd, Iran; ^3^ Clinical Research Center, Department of Internal Medicine, School of Medicine, North Khorasan University of Medical Science, Bojnurd, Iran; ^4^ Department of Pharmaceutics, School of Pharmacy, Shahid Sadoughi University of Medical Sciences and Health Services, Yazd, Iran; ^5^ Department of Clinical Pharmacy, School of Pharmacy, Shahid Sadoughi University of Medical Sciences and Health Services, Yazd, Iran

**Keywords:** mechanical ventilation, weaning, medroxyprogesterone, donepezil, clinical trial

## Abstract

**Background:** Medroxyprogesterone and donepezil could be used as respiratory stimulants in ventilated patients. However, no randomized placebo-controlled trial is available to confirm this approach and compare these drugs. The aim of the current study was to evaluate the effects of donepezil or medroxyprogesterone compared to the placebo in improvement in respiratory status and weaning facilitation in critically ill adult patients receiving mechanical ventilation.

**Material and Methods:** This randomized, triple-blind trial was conducted on 78 ventilated patients in intensive care units (ICU). Patients who were intubated due to pulmonary disorders were ruled out. Patients were randomized in a 1:1:1 ratio to receive 5 mg donepezil (n = 23) or 5 mg medroxyprogesterone (n = 26), or placebo (n = 24) twice a day until weaning (maximum 10 days). The primary endpoints were weaning duration, and duration of invasive mechanical ventilation. Secondary endpoints included rate of successful weaning, changes in arterial blood gas (ABG) parameters, GCS and sequential organ failure assessment (SOFA) score, hemoglobin (Hgb), ICU-mortality, and duration of ICU stay, were measured before and after the intervention and if successful weaning was recorded.

**Results**: Of 78 studied patients who were randomized, 59 weaned successfully. 87% patients in donepezil and 88.5% patients in medroxyprogesterone groups were successfully weaned compared to 66.7% patients in the placebo group. However, this difference was not statistically significant (*p-Value* = 0.111). Changes in pH, mean duration of intubation, and weaning duration were statistically different in donepezil compared with the control group (*p-Value* < 0.05). No significant difference in ABG, Hgb, GCS and SOFA score, and duration of intubation were seen in the medroxyprogesterone group, but weaning duration was significantly reduced to 1.429 days compared with the control group (*p-Value* = 0.038).

**Conclusion:** The results of this clinical trial have demonstrated that the administered dose of medroxyprogesterone and donepezil can expedite the weaning process by reducing the weaning duration compared to placebo. Furthermore, the total duration of invasive ventilation was significantly lower in the donepezil group compared to the control group. Future clinical trials with a larger sample size will determine the exact role of medroxyprogesterone and donepezil in mechanically ventilated patients.

**Clinical Trial Registration:** https://irct.ir/IRCT20190810044500N2 (April 1, 2020).

## Introduction

Mechanical Ventilation (MV) with positive pressure was first introduced clinically in the 1950s during the polio epidemic (Dries and Perry, 2008). Although positive pressure ventilation is one of the most important medical care interventions nowadays and can be vital in cases with indications, duration of intubation correlates with an increased mortality rate (Dries and Perry, 2008; Peñuelas et al., 2011). Because of the ventilator-associated complications, the weaning process should be initiated as soon as possible (McLean et al., 2006).

About 20–30% of ventilated patients are considered difficult to wean from invasive MV (Heunks and Van Der Hoeven, 2010). The time spent in the weaning process may necessitate 40% of the overall duration of MV (Cohen et al., 2019). Difficulty in weaning can be due to reduced central respiratory stimulants, which is often accompanied by the decreased level of consciousness, nervous system damage, and metabolic encephalopathy (Chao and Scheinhorn, 1998).

In spite of insufficient data regarding the efficacy of respiratory stimulants such as caffeine and doxapram (Giguère et al., 2008), researchers are still seeking for new strategies to reduce duration of intubation and treatment costs (Dasta et al., 2005). Available evidence indicates that muscarinic acetylcholine receptors have potent excitatory consequences on medullary respiratory neurons and motoneurones, and are likely to contribute to changes in central hemosensitive drive to the respiratory control system (Yamada and Ichinose, 2018). On one hand the central role of cholinergic mediators in the respiratory system is the basis for its positive effects in neurological disorders related to respiratory control (Abbasi et al., 2015). On the other hand, the parafacial respiratory group plays an important role in the generation of active expiration and recruitment of expiratory abdominal muscles. It is under GABAergic and cholinergic control and is activated in conditions of high metabolic demand (Pisanski and Pagliardini, 2019). Donepezil, a cholinergic drug that reversibly and non-competitively inhibits central acetyl-cholinesterase, has improved cognitive function significantly (Abbasi et al., 2015). As thalamic cholinergic deficit may contribute to obstructive sleep apnea (OSA), donepezil could be a potential modality to improve OSA (Gilman et al., 2003; Moraes et al., 2008; Sukys-Claudino et al., 2012).

Progesterone is a respiratory stimulant improving ventilation function, ventilation capacity, and exercise efficiency in normal individuals and obstructive airways diseases. Progesterone could enhance ventilation, breathing overnight and respiratory gas exchanges (Schoene et al., 1980; Delaunois et al., 1985; Wagenaar et al., 2003; Golparvar et al., 2005; Saaresranta et al., 2005). The lipophilic properties of progesterone allow it to enter the hypothalamic sites that are mediated by estrogen (E2) receptors in the central nerve system (CNS) (Bayliss and Millhorn, 1992). Another proposed mechanism is that progesterone stimulates leptin hormone release, which may increase ventilation (Saaresranta et al., 2002).

The aim of the current study was to evaluate the effects of donepezil or medroxyprogesterone compared to the placebo in improving the respiratory status and weaning facilitation in critically ill adult patients with non-pulmonary etiologies receiving mechanical ventilation for the first time. In addition to weaning duration, and duration of invasive mechanical ventilation as our primary endpoint, we evaluated secondary endpoints, including successful weaning rate from MV, changes in arterial blood gas (ABG) parameters, GCS and sequential organ failure assessment (SOFA) score, hemoglobin (Hgb), ICU-mortality, and duration of intensive care units (ICUs) stay, were measured before and after the intervention and if successful weaning was recorded.

## Methods

### Patients

This randomized triple-blind placebo-controlled clinical trial was conducted in Rahnemoon tertiary referral hospital affiliated to Shahid Sadoughi University of Medical Sciences, which is the neurological referral center of Yazd city. Recruitment was conducted from December 20, 2019, through July 22, 2020.

Inclusion criteria were age older than 18 years, MV through the endotracheal tube for at least 24 h (hrs) prior to study enrollment, oxygen saturation (O_2_Sat) greater than 90%, Fraction of inspired oxygen (FIO_2_) less than 50%, and positive end-expiratory pressure (PEEP) less than 8 cm H_2_O, Hemodynamic stability during the last 12 h, discontinuation of sedatives or reduction of sedation for the past 48 h, neurologic stability with Ramsay score ≤5, and body temperature between 36 and 38 centigrade degrees (°C).

Exclusion criteria were pregnancy or lactation, known allergy to donepezil or medroxyprogesterone, Alzheimer disease, tracheostomy, hepatic encephalopathy, sever cerebral edema, acute hydrocephalus, myasthenia gravis, acute polyneuropathy, prolonged cardiac arrest with poor neurological prognosis, acute right ventricular failure, history of renal failure, blood urea nitrogen (BUN) > 25 mmol/L, plasma creatinine >180 mmol/L or creatinine clearance <30 ml/min, or more than 25% increase in creatinine over the last 24 h; contrast injection in the last 6 hours; serum sodium level >150 mEq/L, serum potassium level <3.5 mEq/L, metabolic alkalosis with arterial pH > 7.5, GCS = 3, and concomitant administration of potential respiratory stimulants. Had not taken donepezil or medroxyprogesterone during the previous 14 days (according to the half-lives of drugs). We also excluded patients with known underlying lung disease as the severity of lung disease can influence the duration and success rate of the weaning.

### Study Design

The anesthesiologist, ward nurses, and the data collector were all blinded to the intervention assignments throughout the study. Patients were randomized in a 1:1:1 ratio to one of three groups including medroxyprogesterone (n = 26) or donepezil (n = 26) or placebo (n = 26) based on a random number table. Before attempting to wean patients from MV, the necessary assessment were performed before conducting a spontaneous breathing trial. For intervention groups, medroxyprogesterone and donepezil were administered 10 mg/day (5 mg/BID) and patients in the control group received the placebo twice daily until weaning and for a maximum of 10 days. Weaning failure was considered as the need for tracheostomy or intervention for more than 10 days.

### Preparation of Placebo Tablets

For preparation of placebo tablets, the determined amount of lactose, microcrystalline cellulose, and stearic acid was weighted exactly, mixed with a tumbler mixer, and directly compressed with a single punch tablet press machine. The hardness and thickness of placebo tablets were adjusted similar to donepezil and medroxyprogesterone tablets. Placebo was prepared in the Pharmaceutics Laboratory of Shahid Sadoughi School of Pharmacy, Yazd. Donepezil and medroxyprogesterone tablets were purchased from Sami Saz and Iran Hormone pharmaceutical companies, respectively.

### Data Gathering

Demographic information, registry numbers, and dates of reception were recorded in the prepared questionnaire. The following variables including ABG (PaO_2_, PaCO_2_, HCO_3_, pH, and O_2_Sat), GCS, Hbg, and SOFA scores were recorded before the intervention, after 24 h, and if successful weaning happened.

### Weaning Procedure

When the patient remains clinically stable, the weaning process starts with assessing the ability of the patient for breath spontaneously. Spontaneous breathing included using continuous positive airway pressure (CPAP), or invasive ventilation with low pressure support, for some cases supplemental oxygen through a T-piece connection applies, If the patient develops signs of poor tolerance, weaning is considered to have failed and mechanical ventilation is reinstituted (Zein et al., 2016).

### Randomization

By applying permuted block randomization, we randomly allocated the eligible patients into one of three arms of the study. For this reason, we used blocks of four. The principal investigator, who was unaware of the interventions, gave one digit number to each prepared formulation from 1 to 78. The first eligible person was given to number 1, the second person as number 2, and so on. The anesthesiologist, the ward nurses, and the data collector were all blinded to the intervention assignments throughout the study. In the end, the prepared formulation were decoded and assigned to the proper arm of the study by the main principal.

### Sample Size

The sample size was estimated as 26 per group based on earlier experience and HCO^−^_3_ standard deviation in donepezil and medroxyprogesterone groups of 0.3 and 7.4, respectively in order to reach a mean difference (reduction in total clinical score) of 4 with the following specifications and using the sample size eq.

$n=\frac{{\left(Z_{\left(1-\frac{\alpha }{2}\right)}+Z_{1-\beta }\right)}^{2}2S^{2}}{{\left(\mu_{1}-\mu_{2}\right)}^{2}},$ for comparing two means; the estimated sample size was increased to 27 per group to take account of potential attrition 10% (*α* = 0.05; *β* = 0.2).

### Statistical Analysis

The quantitative and qualitative variables were reported as mean ± standard deviation (SD) and number (frequency), respectively. The normality of the data was checked by Kolmogorov-Smirnov test. The distributed quantitative variables were compared between groups by using the independent sample *t*-test and Mann-Whitney, respectively. Moreover, paired *t*-test was used to compare changes of variables at groups over 24 h and repeated measurement to compare the changes of variables over time. Chi-square and Fisher’s exact tests were used to test to compare qualitative variables. Two-way ANOVA and General Liner model were applied to evaluate the simultaneous effects of medications, time and variables changes. All the statistical analysis was conducted by Statistical package for social science (SPSS) software version 23 and two-tailed *p*-values < 0.05 were considered statistically significant.

## Results

**FIGURE 1:**
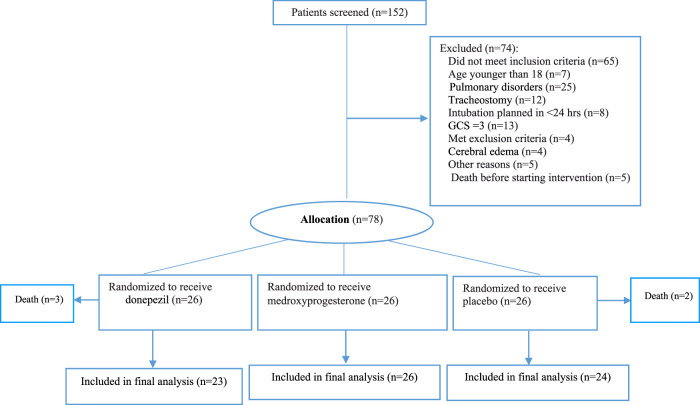
Flowchart of the study.

In total, 152 patients were screened for eligibility in three neurological ICUs of our hospital. We enrolled 78 intubated patients without primary pulmonary diseases. The mean age of the participants was 41.23 ± 24.2 years old. The total intention to treat population were 73 patients who randomly assigned to the following groups: 26 patients each assigned to the control, donepezil, or medroxyprogesterone groups. Five patients were withdrawn from the study subsequently. Eventually, 73 patients completed the study for outcome measurement (Figure 1). Patients in studied groups were balanced in terms of demographic and disease characteristics (Table 1). In order to adjust stroke and head injury covariance analysis was used. By entering these variables into the model, significant difference have been seen between groups (*p-Value* = 0.015).

**TABLE 1:** Characteristics of the patients at baseline.

Parameters	Placebo (N = 24)	Medroxyprogesterone (N = 26)	Donepezil (N = 23)	*p*-value
Age, mean ± SD	39.2 ± 21	45.5 ± 20.8	38.7 ± 22.1	0.483
Men, no. (%)	20 (83.3)	21 (80.8)	18 (78.2)	0.828
SOFA scores, mean ± SD (points)	7.6 ± 1.7	6.7 ± 1.7	7.1 ± 2.1	0.237
ICU stay before randomization, mean ± SD (h)	54.0 ± 58.8	50.7 ± 31.3	45.9 ± 27.9	0.800
Coexisting conditions, no (%)				
Hypertension	6 (25)	5 (19.2)	3 (13)	0.582
Diabetes mellitus	0 (0.0)	0 (0.0)	1 (4.34)	0.332
Dyslipidemia	0 (0.0)	1 (3.8)	0 (0.0)	0.400
Hyperactive delirium, no (%)	1 (4.1)	2 (7.6)	1 (4.3)	0.365
Causes of invasive mechanical ventilation, no. (%)				
Stroke	9 (37.5)	17 (65.3)	12 (52.1)	0.147
Surgery	4 (16.6)	2 (7.6)	4 (17.3)	0.550
Head trauma	4 (16.6)	1 (3.8)	2 (8.6)	0.311
Brain edema	2 (8.3)	1 (3.8)	3 (13.0)	0.515
Cerebral contusion	2 (8.3)	1 (3.8)	1 (4.3)	0.761
Others	3 (12.5)	4 (15.3)	1 (4.3)	0.379
Sedative, opioid and other CNS depressant medications, No. (%)				
Midazolam	24 (100)	26 (100)	23 (100)	1.000
Fentanyl	24 (100)	26 (100)	23 (100)	1.000
Morphine	15 (62.5)	18 (69.2)	18 (78.2)	0.498
Methadone	2 (8.3)	1 (3.8)	1 (4.3)	0.753
Propofol	1 (4.1)	0 (0.0)	1 (4.3)	0.566
Thiopental	1 (4.1)	1 (3.8)	0 (0.0)	0.622
Other medications, No. (%)				
Antibiotic				
Carbapenem	0	2 (7.6)	1 (4.3)	0.422
Cephalosporins	16 (66.6)	21 (80.7)	14 (60.8)	0.413
Glycopeptide	5 (0)	5 (19.2)	2 (8.6)	0.457
Aminoglycoside	2 (8.3)	4 (15.3)	4 (17.3)	0.676
Clindamycin	6 ()	4 (15.3)	3 (13)	0.443
Penicillins	1 (4.1)	1 (3.8)	0	0.617
Cardiovascular				
Antiplatelet	2 (8.3)	3 (11.5)	0	0.276
Anticoagulant	12 (50)	10 (38.4)	9 (39.1)	0.497
ACEI/ARB	3 (12.5)	2 (7.6)	3 (13)	0.752
CCB	1 (4.1)	3 (11.5)	1 (4.3)	0.547
Β-Blockers	5 (20.8)	6 (23)	3 (13)	0.666
Nitrate	0	1 (3.8)	0	0.424
Diuretics	6 (25)	7 (26.9)	3 (13)	0.461
Anticonvulsant				
Phenytoin	7 (29.1)	5 (19.2)	6 (26)	0.598
Valproate sodium	2 (8.3)	4 (15.3)	4 (17.3)	0.688
Gastrointestinal				
H2B/PPI	20 (83.3)	25 (96.1)	18 (78.2)	0.253
Prokinetic agents	5 (20.8)	8 (30.7)	4 (17.3)	0.586
Others				
Corticosteroids	8 (33.3)	7 (26.9)	5 (21.7)	0.589
NSAIDs/Acetaminophen	10 (41.6)	8 (30.7)	5 (21.7)	0.274
Ascorbic acid	7 (29.1)	9 (34.6)	10 (43.4)	0.687
Insulin	2 (8.3)	0	1 (4.3)	0.300
Laboratory measurements at inclusion, Mean ± SD				
pH	7.37 ± 0.09	7.37 ± 0.84	7.36 ± 0.05	0.789
PaO_2,_ mm Hg	130.0 ± 48.7	137.1 ± 50.8	131.0 ± 52.4	0.867
PaCO_2,_ mm Hg	38.9 ± 8.8	37.3 ± 11.3	40.4 ± 7.1	0.523
HCO_3_, mEq/L	22.8 ± 4.4	20.8 ± 5.2	21.8 ± 3.6	0.331
Creatinine, mg/dL	1.2 ± 0.6	1.3 ± 1.0	1.0 ± 0.4	0.402
Bilirubin, mg/dL	0.8 ± 0.3	0.8 ± 0.1	0.7 ± 0.4	0.252
Hemoglobin, g/dL	10.9 ± 2.5	11.2 ± 2.0	11.7 ± 1.8	0.608
Blood platelets, ×10^3^/μL	140.6 ± 59.4	167.7 ± 71.2	144.8 ± 49.0	0.244

No, Number; hrs, hours; SD, standard deviation; pH, potential of hydrogen; PaO_2_, partial pressure of oxygen, arterial; PaCO_2_, partial pressure of carbon dioxide; mm Hg, millimeter of mercury; HCO_3_; Bicarbonate; mEq/L, mill equivalents per liter; mg/dL, milligrams per deciliter; g/dL, grams per deciliter; μL, microliter; Chi-square were used to compare these values.

### Primary Outcomes

The mean (SD) of total duration of invasive ventilation was 142.8 (47.4) hrs in the medroxyprogesterone and 124.8 (51.36) hrs in the donepezil groups vs. 176.4 (64.8) hrs in the control group, for a between-group difference of 33.6 h for medroxyprogesterone group and 51.6 h for donepezil compared to the control group. As presented in Table 2, the mean total duration of invasive ventilation was significantly lower in the donepezil compared to the control group (*p-Value* = 0.02).

**TABLE 2:** Trends of changes in primary outcomes.

Variables	Comparing		Between-group mean difference	*p*-value
Duration of invasive mechanical ventilation, hours	placebo	Medroxyprogesterone	+33.600	0.161
		donepezil	+51.768	**0.020** ^a^
	Medroxyprogesterone	donepezil	+18.144	0.511
Weaning duration, hours	placebo	Medroxyprogesterone	+34.296	**0.038** ^a^
		donepezil	+42.600	**0.010** ^a^
	Medroxyprogesterone	donepezil	+8.280	0.793

a Statistically significant (*p-Value < 0.05*). ANOVA and Tukey HSD, tests were used to compare these value**s**.

For the medroxyprogesterone, compared to the donepezil group, no significant between-group differences were found for the mean duration of weaning (64.5–83.2–56.4 h, *p*-value = 0.793). The duration of weaning was a mean (SD) of 98.8 (46.8) hrs in the control group vs. 64.5 (42.96) hrs and 56.4 (35.76) hrs for medroxyprogesterone and donepezil respectively, for a between-group difference of 34 and 42.4 h in the donepezil and medroxyprogesterone groups, respectively. This value did significantly in the groups (*p-Value* < 0.05) (Table 2).

### Secondary Outcomes

From 73 enrolled patients who were randomized in 1:1:1 ratio to one of three groups, 16 (66.7%) patients in control, 23 (88.5%) patients in medroxyprogesterone, and 20 (87%) patients in the donepezil group experienced successful weaning during the study period. However, the rate of successful weaning were greater in the donepezil and medroxyprogesterone compared to the control group this difference was not statistically significant by Chi-Square test (*p-Value* = 0.111).

The comparison between group differences was done using the ANOVA and Tukey HSD test. In successfully weaned patients, the total duration of ICU stay was the mean (SD) of 453.6 (232.8) hrs in the medroxyprogesterone and 352.8 (180) hrs in the donepezil groups vs. 434.4 (170.4) hrs in the control group, for a between-group difference of −19.68 and 79.2 h for the medroxyprogesterone and donepezil groups respectively. However, this value did not differ significantly between the groups (*p-Value* = 0.245). The rate of ICU mortality was not different between groups, as 4 (25%) patients in control, 5 (21.7%) in medroxyprogesterone, and 3 (15%) patients in the donepezil group expired during study follow-up (*p-Value* = 0.245).

**FIGURE 2:**
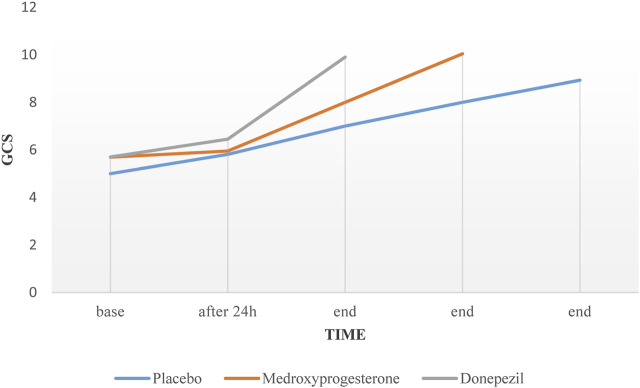
Mean variations of GCS in three different groups over the time.

In patients with successful weaning, percentage of pH changes in the donepezil group was significant compared to the control group (*p-Value* = 0.048). Despite the upward trend in the profiles of the GCS over time in all three groups, no significant difference was observed between groups (*p-Value* = 0.084) (Figure 2).

In non-successfully weaned patients, the total duration of ICU stay was on average 559.2 (276) and 758.4 ( 278) hrs in the medroxyprogesterone and donepezil groups in comparison to 532.8 (232.8) hrs in the control group, for a between-group difference of −24.0 h and −223.2 h, respectively. This value did not change significantly in the groups (*p*-value = 0.453). The rate of ICU mortality in non-successfully weaned patients was 2 (25%) in control, and no patient in other groups (*p-Value* = 0.480).

Moreover, the comparison other secondary endpoints, including arterial blood gas (ABG) parameters, O2Sat, Hgb, and SOFA score was done using the general linear model test. The results did not differ significantly after 24 h in non-successfully and overtime in successfully weaned patients. There were no significant differences in mean difference of variables between each group compared to the others (Tables 3 and 4).

**TABLE 3:** Secondary outcomes in successfully weaned patients before, after 24 h, and when weaning happened.

Variables	Group, mean ± SD									*p*-value
	Placebo (N = 16)			Medroxyprogesterone (N = 23)			Donepezil (N = 20)			
Successful weaning, No. (%)	16 of 24 (66.7)			23 of 26 (88.5)			20 of 23 (87)			0.111
ICU stay, hrs	434.4 ± 170.4			453.6 ± 232.8			352.8 ± 180			0.245
ICU mortality, No. (%)	4 (25)			5 (21.7)			3 (15)			0.753
	before	24 h	AW	before	24 h	AW	before	24 h	AW	
ABG parameters										
O_2_Sat, %	96.5 ± 1.9	97.5 ± 1.5	97.5 ± 1.8	97.2 ± 1.4	96.9 ± 3.5	97.2 ± 1.7	97.4 ± 2.1	97.8 ± 1.2	96.8 ± 1.9	0.726
PaO_2_, mm Hg	129.4 ± 36.9	114.5 ± 15.5	118.9 ± 33.9	134.4 ± 52.3	130.7 ± 39.5	113.3 ± 16.6	132.2 ± 55.5	121.5 ± 31.6	106.3 ± 29.0	0.655
PaCO_2_, mm Hg	38.6 ± 10.0	35.4 ± 12.3	36.0 ± 6.1	36.9 ± 12.0	35.7 ± 12.3	35.4 ± 5.7	40.4 ± 7.6	36.0 ± 8.3	35.2 ± 4.8	0.460
HCO_3_, mEq/L	22.1 ± 3.6	5.2 ± 21.9	23.2 ± 3.7	20.6 ± 5.4	22.6 ± 4.8	24.1 ± 3.0	26.0 ± 4.5	22.2 ± 3.8	23.2 ± 4.0	0.655
pH	7.37 ± 0.10	7.41 ± 0.07	7.42 ± 0.08	7.37 ± 0.08	7.39 ± 0.11	7.40 ± 0.05	7.36 ± 0.05	7.39 ± 0.05	7.41 ± 0.05	**0.048** ^a^
Hemoglobin, g/dl	10.9 ± 2.5	10.7 ± 2.4	10.3 ± 1.8	11.2 ± 2.0	10.7 ± 1.9	10.5 ± 2.3	11.7 ± 1.8	11.1 ± 2.0	11.1 ± 1.8	0.143
GCS	5.0 ± 0.9	5.8 ± 1.1	8.9 ± 0.7	5.6 ± 1.2	5.9 ± 1.6	10.0 ± 1.1	5.7 ± 1.2	6.4 ± 1.7	9.9 ± 1.2	0.084
SOFA scores, points	7.9 ± 1.8	7.1 ± 1.7	3.8 ± 1.2	6.7 ± 1.8	8.1 ± 1.9	3.2 ± 1.0	6.9 ± 2.1	8.1 ± 1.6	3.3 ± 1.2	0.655

No, number; SD, standard deviation; ABG, arterial blood gas; pH, potential of Hydrogen; PaO_2_, partial pressure of oxygen; PaCO_2_, partial pressure of carbon dioxide; mm Hg, millimeter of mercury; HCO_3_; Bicarbonate; mEq/L, mill equivalents per liter; mg/dl, milligrams per deciliter; g/dl, grams per deciliter; GCS, glasgow coma scale; SOFA, sequential organ failure assessment score; AW, after weaning.

a Statistically significant (*p-Value < 0.05*)

**TABLE 4:** Secondary outcomes in non-successfully weaned patients before and after 24 h.

Variables	Group, mean ± SD									*p*-value
	Placebo (N = 8)			Medroxyprogesterone (N = 3)			Donepezil (N = 3)			
ICU stay, hrs	532.8 ± 218.4			559.2 ± 276			758.4 ± 276			0.430
ICU mortality, No. (%)	2 (25)			0			0			0.480
	before	24 h		before	24 h		before	24 h		
ABG parameters										
O_2_Sat, %	94.8 ± 6.8		98.1 ± 0.9	97.3 ± 2		98 ± 0	96 ± 1		96.6 ± 1.5	0.220
PaO_2_, mm Hg	131.3 ± 69.7		124.7 ± 37.6	157.6 ± 37.2		130.6 ± 30	123 ± 29.5		114 ± 27.4	0.930
PaCO_2_, mm Hg	39.3 ± 6.4		37.3 ± 6.2	40.3 ± 3		30.6 ± 6.1	40.6 ± 1.5		39.3 ± 6.8	0.719
HCO_3_, mEq/L	24.1 ± 5.8		24 ± 3.2	22.8 ± 3.2		21 ± 5.3	22.4 ± 2.9		24.4 ± 2.1	0.367
pH	7.38 ± 0.77		7.38 ± 0.67	7.37 ± 0.72		7.45 ± 0.06	7.35 ± 0.37		7.42 ± 0.02	0.941
Hemoglobin, g/dl	11.1 ± 2.7		10.3 ± 2.4	12.1 ± 1.4		11.7 ± 0.6	11.5 ± 1.2		11.5 ± 0.8	0.540
GCS	5.5 ± 1.1		5.2 ± 0.7	4.6 ± 0.5		5 ± 0	4.6 ± 1.1		4.6 ± 0.57	0.569
SOFA scores, points	7 ± 1.3		8.1 ± 2.4	6.3 ± 0.5		8.3 ± 2	8.6 ± 1.5		8 ± 1.7	0.199

No, number; SD, standard deviation; ABG, arterial blood gas; pH, potential of Hydrogen; PaO_2_, partial pressure of oxygen; PaCO_2_, partial pressure of carbon dioxide; mm Hg, millimeter of mercury; HCO_3_; Bicarbonate; mEq/L, mill equivalents per liter; mg/dl, milligrams per deciliter; g/dL, grams per deciliter; GCS, glasgow coma scale; SOFA, sequential organ failure assessment score.

## Discussion

Although, the effects of hormones such as progesterone on respiratory and ventilatory performance have been evaluated in previous studies, the present study is the first randomized, controlled clinical trial evaluating the effectiveness of medroxyprogesterone in facilitation of weaning in patients receiving invasive mechanical ventilation. The effect of donepezil was previously assessed in a clinical study to facilitate the weaning course. However, in this non-randomized study, there was no control group and the effect of donepezil on ABG was evaluated in only 16 difficult to wean patients. According to the design of this aforementioned study, it had all the limitations of non-clinical trial studies. Therefore, this study is also the first randomized clinical trial of donepezil in expedition the weaning course in mechanically ventilated patients.

This triple-blind randomized clinical trial designed to compare the improvement in respiratory status and weaning facilitation in mechanical ventilated critically ill adult patients receiving donepezil or medroxyprogesterone vs. placebo. The mean total duration of invasive ventilation was significantly lower in the donepezil compared to the control group while the duration of weaning did differ significantly between the groups. In addition, the rate of successful weaning were greater in the donepezil and medroxyprogesterone groups compared to the control group, this difference was not statistically significant. Donepezil and medroxyprogesterone had no significant effect on the total duration of ICU stay, rate of ICU mortality, and GCS in successfully weaned patients which was not too far from expected, since there are several factors in the ICU setting influencing these parameters and it is not possible to control for all these confounders. In fact, parameters such as age, chronic use of corticosteroid, atrophy of diaphragmatic, or severe limitation of airway flow are far more vigorous factors affecting the successful rate of weaning from mechanical ventilation or outcomes of critically ill patients (Groenewegen et al., 2003; Boles et al., 2007; Levine et al., 2008). Taken together, although patients in donepezil and medroxyprogesterone groups were more successful than the control group in the weaning process, this difference was not statistically significant.

Mechanical ventilation is implemented to improve oxygen delivery and ventilation, and decrease the breathing workload. It is by the physician to and is therefore a means of treating the patient. Physicians adopt MV to stabilize the patient until the diagnosis and therapeutic management of the disease is performed (Boles et al., 2007). In other words, MV is a means to an end and not a treatment in itself. Therefore, the weaning process should be initiated as soon as possible (McLean et al., 2006).

Despite the salvage of MV in critically ill patients, MV is associated with numerous complications. The most common complications are pneumothorax, broncho pleural fistula, nosocomial pneumonia, and respiratory alkalosis, which can impair cerebral perfusion, predispose patients to cardiac dysrhythmias, and prolong the weaning process (Groenewegen et al., 2003). Difficulty in weaning can be due to reduced central respiratory stimulants that suggesting new strategies to reduce the duration of intubation and treatment costs.

Some other agents such as methylphenidate, benzquinamide, ethamivan, bemegride, nikethamide, naloxone, almitrin, theophylline, methyl xanthine, levosimendan, and acetazolamide have been tried as the respiratory stimulants (Dallemagne and Heymans, 1955; Yost, 2006; Roesthuis et al., 2019), but the results of these studies were not encouraging.

The efficacy of acetazolamide in the duration of intubation was evaluated as regards the results did not show reduction in intubation and weaning process (Faisy et al., 2016).

Donepezil and medroxyprogesterone at dose of 10 mg are well tolerated without causing any life-threatening side effect (Soufir et al., 2011; Tan et al., 2014). Mechanically ventilated patients with potential difficulties in respiratory drive were enrolled. Due to the stimulatory properties of progesterone in the central respiratory system and the role of the cholinergic system in the neurological control of respiration, this study was performed. Administration of oral medroxyprogesterone or donepezil in the clinical setting for weaning from MV is limited. No adverse effects were seen in either group.

Medroxyprogesterone could reduce the duration of intubation significantly. We monitored respiratory ventilation function by obtaining serial ABG samples. However, no significant difference observed in all variables of ABG after 24 h and after successful weaning in the medroxyprogesterone group compared with the control group. Contrary to the results of the present study, two previous studies that were conducted on patients with chronic obstructive pulmonary disease (COPD) illustrated the effective role of medroxyprogesterone in PaCO_2_ reduction and PO_2_ increment (Delaunois et al., 1985; Wagenaar et al., 2003). It should be borne in mind that the dosing of medroxyprogesterone was lower in the current study.

In an interventional pilot study, the effect of donepezil on the facilitation of weaning in difficult to wean patients was evaluated. Donepezil was administered for eligible patients at the dose of 10 mg daily for 2–4 weeks. It was presumed that donepezil could expedite weaning by stimulation of respiratory center and prevent the need for re-intubation in these patients. However, they included a small number of patients (16 patients) as well as non-randomization limited the establishment of a strong relationship between use of donepezil and facilitation of weaning (Abbasi et al., 2015). Our results showed that donepezil was able to facilitate the patient’s weaning but did not alter PaO_2_, PaCO_2_, HCO_3_ concentration, and O_2_Sat parameters while contrary to Abbasi´s study pH changes were significant in this study. Although no significant different in pH changes were seen in the donepezil compared to control group after 24 h, changes in pH were significant overtime indicating that several doses of the donepezil are required for an efficient response. It can be argued that the results of ABG parameters such as pH will often influence the weaning process accompanied with better clinical outcome in such patients (Kraut and Madias, 2012).

We also evaluated the effect of donepezil and medroxyprogesterone on the improvement of the patient’s GCS. In the clinical setting, the improvement in GCS is one of the important criteria for initiation of weaning. In other words, low GCS at the beginning of the weaning process is considered a major reason for prolongation of this process (Pu et al., 2015). In this study, despite the increase in GCS over time, this difference was not statistically significant when compared to control group. but this issue, especially in the case of donepezil, requires study in larger populations and longer time because the cholinergic system is one of the most important cognitive centers in the brain (Zhang et al., 2004).

SOFA is a valuable score for the management of patients in ICUs which predicts patients’ outcomes (Ferreira et al., 2001). GCS and PaO_2_ are two main indices for calculation of SOFA score. Due to the lack of changes in GCS and PaO_2_ in both groups compared to placebo, changes in this index were not significant between intervention and placebo groups, so these two drugs are not effective in reducing the SOFA score and mortality rate.

Duration of weaning was significantly reduced in intervention groups compared to the control group. The underlying mechanisms for donepezil are presumed to be due to the performance of the cholinergic system in improving respiratory coordination, increased neuromuscular transmission, improved upper respiratory muscle function, neuronal control of respiration, and increased salivary secretion (Hedner et al., 2003; Proctor and Carpenter, 2014). The performance of medroxyprogesterone in strengthening the tidal volume (VT), respiratory effort, changes in metabolic rate, lung and chest movements, increase ventilation performance and increasing ventilation capacity (Golparvar et al., 2005; Dempsey et al., 2011).

This study has several limitations. First, the main limitation was the small size of the subject groups. Despite more than 7-month study duration and inclusion of consecutive patients, only limited number of patients entered the study according to inclusion and exclusion criteria. Second, while the dose range of medroxyprogesterone was different in previous data, the effective dose for weaning from MV is yet to be determined. Although the interventions performed in this study were safe and no side effects related to these drugs were reported during the study period, but considering the relative short length of the intervention and follow-up period, the necessary precautions should be taken in this regard. Furthermore, the lack of a fixed and predefined protocol for all patients for whom weaning is to be attempted is another limitation which should be taken into consideration in future studies. The last limitation is the presence of factors more strongly influencing the discontinuation of MV or ICU outcomes (eg, age, diagnosis, long-term use of steroids or diaphragm atrophy).

Various population pharmacokinetic parameters and contribution of different factors in weaning process might be the reasons for medroxyprogesterone or donepezil treatment to achieve its response goal during the studies. Therefore, these results require further confirmation with larger controlled clinical trials to clarify and establish medroxyprogesterone and donepezil role on weaning process. Moreover, other confounding factors that strongly influencing the patients’ outcome should be considered.

## Conclusion

Previous investigation found that some respiratory stimulants could facilitate weaning presses. Medroxyprogesterone and donepezil are two safe and successful respiratory stimulants, suggesting that they could be considered as a weaning-facilitating strategy. Our results illustrated that the administered dose of medroxyprogesterone and donepezil can expedite the weaning process presumably by central stimulation of the respiratory system.

The results of the current study have demonstrated that administering medroxyprogesterone or donepezil in the weaning process significantly reduced the weaning duration compared to placebo, while the total duration of invasive ventilation was significantly lower in the donepezil group compared to the control group. There were no statistically significant differences in other evaluated outcomes except pH changes. Although the findings of the present study are interesting, care should be taken in implementing these results.

## Data Availability

The raw data supporting the conclusions of this article will be made available by the authors, without undue reservation.

## Glossary

ANOVA: Analysis of variance
ABG: arterial blood gas
BUN: blood urea nitrogen
cm H_2_O: centimeters of water
C: centigrade degrees
CI: Confidence Interval
COPD: chronic obstructive pulmonary disease.
CNS: central nervous system
FIO_2_: Fraction of inspired oxygen
GCS: glasgow coma scale
hr: hours
mg: milligram
Hbg: hemoglobin
ICU: intensive care unit
MV: Mechanical Ventilation
O_2_Sat: oxygen saturation
OSA: obstructive sleep apnea
pH: potential of hydrogen
SOFA: sequential organ failure assessment
PEEP: positive end-expiratory pressure
mmol/L: mill moles per liter
mEq/L: mill equivalents per liter
ml/min: mill per minute
BID: twice a day
PaO_2_: partial pressure of oxygen
PaCO_2_: partial pressure of carbon dioxide
HCO3: Bicarbonate
mm Hgmillimeter of mercury mg/dL: millimeter of mercury mg/dLmilligrams per deciliter
g/dL: grams per deciliter
SPSS: Statistical package for social science
MD: mean difference
IQR: Inter Quartile Range
